# (Methanol-κ*O*)bis­{2-meth­oxy-6-[(4-methyl­phen­yl)iminiometh­yl]phenolato-κ^2^ *O*,*O*′}tris­(nitrato-κ^2^ *O*,*O*′)cerium(III)

**DOI:** 10.1107/S1600536810026139

**Published:** 2010-07-14

**Authors:** Jia-Lu Liu, Jian-Feng Liu, Guo-Liang Zhao

**Affiliations:** a College of Chemistry and Life Sciences, Zhejiang Normal University, Jinhua 321004, People’s Republic of China, and, Zhejiang Normal University Xingzhi College, Jinhua 321004, People’s Republic of China

## Abstract

The asymmetric unit of title compound, [Ce(NO_3_)_3_(C_15_H_15_NO_2_)_2_(CH_3_OH)], consists of two Schiff base 2-meth­oxy-6-[(4-methyl­phen­yl)iminiometh­yl]phenolate (H*L*) ligands, three nitrate anions and a methanol ligand. The Ce^III^ ion is 11-coordinated: three nitrate radical anions coordinate to the Ce^III^ ion through O atoms, two H*L* ligands chelate the Ce^III^ ion through the O atoms of the phenolate and meth­oxy groups, and one methanol mol­ecule coordinates to Ce^III^ ion through its O atom. The O atom of one nitrate anion is disordered over two sites of equal occupancy. The protonated imine N atoms are involved in intra­molecular hydrogen bonds with the phenoxide groups. C—H⋯O inter­actions are also observed.

## Related literature

For related structures, see: Li *et al.* (2008 ▶); Liu *et al.* (2009 ▶); Xian *et al.* (2008 ▶); Zhao *et al.* (2007 ▶).
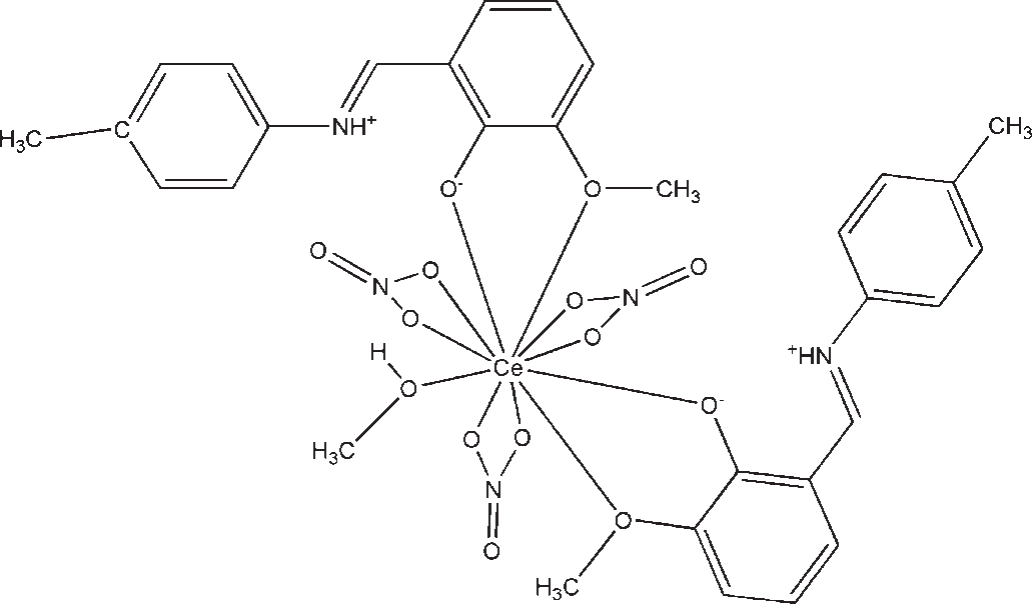


## Experimental

### 

#### Crystal data

[Ce(NO_3_)_3_(C_15_H_15_NO_2_)_2_(CH_4_O)]
*M* *_r_* = 840.75
Triclinic, 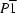

*a* = 7.8540 (2) Å
*b* = 14.6241 (4) Å
*c* = 16.6170 (4) Å
α = 73.0650 (1)°
β = 85.4910 (1)°
γ = 79.3750 (1)°
*V* = 1793.89 (8) Å^3^
*Z* = 2
Mo *K*α radiation
μ = 1.34 mm^−1^
*T* = 296 K
0.46 × 0.18 × 0.17 mm

#### Data collection

Bruker APEXII area-detector diffractometer
Absorption correction: multi-scan (*SADABS*; Sheldrick, 1996 ▶) *T*_min_ = 0.745, *T*_max_ = 0.799
22987 measured reflections
6333 independent reflections
5623 reflections with *I* > 2σ(*I*)
*R*_int_ = 0.051

#### Refinement

*R*[*F* ^2^ > 2σ(*F* ^2^)] = 0.035
*wR*(*F* ^2^) = 0.101
*S* = 1.07
6285 reflections
473 parameters
H atoms treated by a mixture of independent and constrained refinement
Δρ_max_ = 1.06 e Å^−3^
Δρ_min_ = −0.73 e Å^−3^

### 

Data collection: *APEX2* (Bruker, 2006 ▶); cell refinement: *SAINT* (Bruker, 2006 ▶); data reduction: *SAINT*; program(s) used to solve structure: *SHELXS97* (Sheldrick, 2008 ▶); program(s) used to refine structure: *SHELXL97* (Sheldrick, 2008 ▶); molecular graphics: *SHELXTL* (Sheldrick, 2008 ▶); software used to prepare material for publication: *SHELXTL*.

## Supplementary Material

Crystal structure: contains datablocks I, global. DOI: 10.1107/S1600536810026139/pv2292sup1.cif

Structure factors: contains datablocks I. DOI: 10.1107/S1600536810026139/pv2292Isup2.hkl

Additional supplementary materials:  crystallographic information; 3D view; checkCIF report

## Figures and Tables

**Table 1:** Hydrogen-bond geometry (Å, °)

*D*—H⋯*A*	*D*—H	H⋯*A*	*D*⋯*A*	*D*—H⋯*A*
C7—H7*A*⋯O12^i^	0.93	2.51	3.233 (6)	135
C7—H7*A*⋯O14^i^	0.93	2.57	3.488 (5)	169
C30—H30*B*⋯O11^ii^	0.96	2.60	3.405 (6)	142
C13—H13*A*⋯O8^iii^	0.93	2.42	3.304 (5)	160
C22—H22*A*⋯O10^iv^	0.93	2.39	3.243 (5)	153
C29—H29*A*⋯O11^iv^	0.93	2.42	3.285 (7)	154
C10—H10*A*⋯O9	0.93	2.57	3.410 (5)	150
C25—H25*A*⋯O8	0.93	2.52	3.411 (7)	160
N1—H1*A*⋯O1	0.86	2.02	2.671 (4)	132
N1—H1*A*⋯O9	0.86	2.50	3.290 (4)	154
N2—H2*A*⋯O3	0.86	1.96	2.638 (4)	135
N2—H2*A*⋯O7	0.86	2.65	3.444 (6)	154

Symmetry codes: (i) 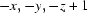; (ii) 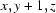; (iii) 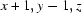; (iv) 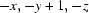.

**Fig. 1.**
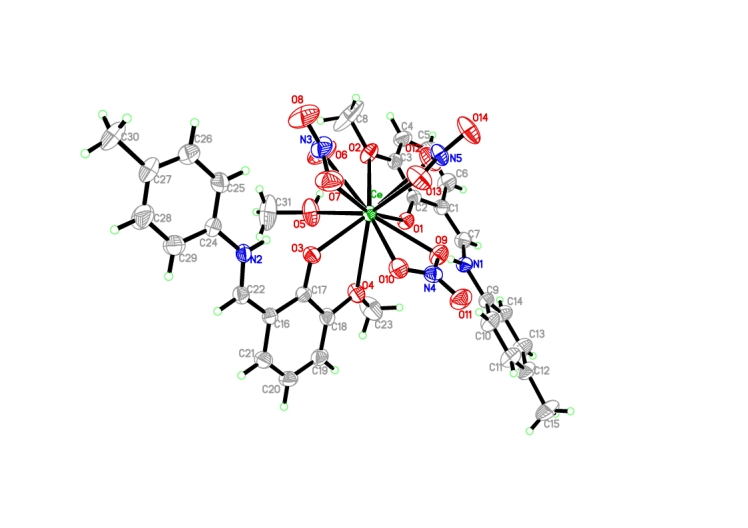
The molecular structure of the title complex, showing the atom-labelling scheme. Displacement ellipsoids are drawn at the 30% probability level.

## supplementary crystallographic information

### Comment

Schiff base complexes have been received much attention for many years. By introducing diverse groups of different shapes and functions, Schiff bases also have potential applications in material science, biological, encapsulation, hydrometallurgy, *et al.* O-vanillin derived Schiff base complexes have been absorbed considerable attention in the past decades due to the intriguing biological activities of *o*-vanillin and the convenience in Schiff bases synthesis. Interested in this field, we have been synthesized several analogous Schiff bases derived from *o*-vanillin and prepared their transitional and rare metal complexes further. In a few of articles we have reported our partial research results (Zhao *et al.*, 2007; Xian *et al.*, 2008; Li *et al.*, 2008). Herein, we describe a new Ce^III^ complex.

The structure of complex (1) was shown in Fig.1 and the coordination environment of Ce^III^ was shown in Fig. 2. In this complex the Ce^III^ is twelve-coordinated by eleven oxygen atoms, six 0 from three nitrate radical ions and four O atoms from the Schiff bases (HL), another one oxygen atom from methanol, which can be described as a distorted sphere. HL ligands coordinate to the Ce^III^ion with bidentate-chelate mode using oxygen atoms from deprotonated phenolic hydroxyl groups and methoxyl groups. The Ce—O bond distances were listed in Table 1, The distances between Ce^III^ and methoxy O atoms are obvious longer than Ce—O(phenolic) bond distances, which are similar to the analogous complexes(Zhao *et al.*, 2007; Li *et al.*, 2008, Liu *et al.*, 2009). The nitrate radical anions coordinate to the Ce^III^ with O atoms with the distances from 2.540–2.733 Å, which are between the Ce—O(phenolic) and the Ce—*O*(methoxy). The Ce–O (methyl alcohol)is only slightly longer than the Ce—O(phenolic).

The hydrogen bonds and π···π weak non-covalent interactions lend stability to the structure. In the structure, In HL ligand, two protons of phenolic hydroxyl groups considered to have transferred to imine N atoms involve in forming intramolecular hydrogen bonds. There are no classic hydrogen bonds between the adjacent molecules, The π···π interactions exist both intra and extra molecules between the approximate paralleled participating benzene rings, which may be the primary forces keep the complex molecules packing together.

### Experimental

Reagents and solvents used were of commercially available quality and without purified before using. The Schiff base ligand 2-[(4- methylphenyl)iminomethyl]-6-methoxy-phenol was synthesized from condensation of *o*-vanillin and *p*-methylaniline. The title compound was synthesized by traditional method. 480 milligram (2 mmol) HL ligand was dissolved in 20 ml me thanol, then 326 milligram (1 mmol) Ce(NO_3_)_3_ (in methanol) was added to the upper solution. The mixture solution was stirred for 2 h at room temperature. At last, deposit was filtered out and the red solution was kept in the open air. The red crystal was obtained after several days.

### Refinement

The structure was solved by direct methods and successive Fourier difference synthesis. The H atoms bonded to C and N atoms were positioned geometrically and refined using a riding model [aliphatic C—H =0.96 Å (*U*_iso_(H) = 1.5*U*_eq_(C)), aromatic C—H = 0.93 Å (*U*_iso_(H) = 1.2*U*_eq_(C)) and N—H = 0.86 Å, *U*_iso_(H) = 1.2*U*_eq_(C)].

### Crystal data

**Table:**

[Ce(NO_3_)_3_(C_15_H_15_NO_2_)_2_(CH_4_O)]	*Z* = 2
*M**_r_* = 840.75	*F*(000) = 850
Triclinic, *P*1	*D*_x_ = 1.556 Mg m^−^^3^
Hall symbol: -P 1	Mo *K*α radiation, λ = 0.71073 Å
*a* = 7.8540 (2) Å	Cell parameters from 9995 reflections
*b* = 14.6241 (4) Å	θ = 1.5–25.0°
*c* = 16.6170 (4) Å	µ = 1.34 mm^−^^1^
α = 73.0650 (1)°	*T* = 296 K
β = 85.4910 (1)°	Block, red
γ = 79.3750 (1)°	0.46 × 0.18 × 0.17 mm
*V* = 1793.89 (8) Å^3^	

### Data collection

**Table:**

Bruker APEXII area-detector diffractometer	6333 independent reflections
Radiation source: fine-focus sealed tube	5623 reflections with *I* > 2σ(*I*)
graphite	*R*_int_ = 0.051
ω scans	θ_max_ = 25.0°, θ_min_ = 1.5°
Absorption correction: multi-scan (*SADABS*; Sheldrick, 1996)	*h* = −9→9
*T*_min_ = 0.745, *T*_max_ = 0.799	*k* = −16→17
22987 measured reflections	*l* = −19→19

### Refinement

**Table:**

Refinement on *F*^2^	Primary atom site location: structure-invariant direct methods
Least-squares matrix: full	Secondary atom site location: difference Fourier map
*R*[*F*^2^ > 2σ(*F*^2^)] = 0.035	Hydrogen site location: inferred from neighbouring sites
*wR*(*F*^2^) = 0.101	H atoms treated by a mixture of independent and constrained refinement
*S* = 1.07	*w* = 1/[σ^2^(*F*_o_^2^) + (0.0645*P*)^2^ + 0.3185*P*] where *P* = (*F*_o_^2^ + 2*F*_c_^2^)/3
6285 reflections	(Δ/σ)_max_ = 0.001
473 parameters	Δρ_max_ = 1.06 e Å^−^^3^
0 restraints	Δρ_min_ = −0.73 e Å^−^^3^

### Special details

**Table:**

Geometry. All e.s.d.'s (except the e.s.d. in the dihedral angle between two l.s. planes) are estimated using the full covariance matrix. The cell e.s.d.'s are taken into account individually in the estimation of e.s.d.'s in distances, angles and torsion angles; correlations between e.s.d.'s in cell parameters are only used when they are defined by crystal symmetry. An approximate (isotropic) treatment of cell e.s.d.'s is used for estimating e.s.d.'s involving l.s. planes.
Refinement. Refinement of *F*^2^ against ALL reflections. The weighted *R*-factor *wR* and goodness of fit *S* are based on *F*^2^, conventional *R*-factors *R* are based on *F*, with *F* set to zero for negative *F*^2^. The threshold expression of *F*^2^ > σ(*F*^2^) is used only for calculating *R*-factors(gt) *etc*. and is not relevant to the choice of reflections for refinement. *R*-factors based on *F*^2^ are statistically about twice as large as those based on *F*, and *R*- factors based on ALL data will be even larger.

### Fractional atomic coordinates and isotropic or equivalent isotropic displacement parameters (Å^2^)

**Table:**

	*x*	*y*	*z*	*U*_iso_*/*U*_eq_	Occ. (<1)
Ce	−0.00955 (2)	0.326368 (13)	0.305560 (12)	0.04189 (10)	
C1	0.2417 (5)	0.0393 (3)	0.5041 (2)	0.0518 (9)	
C2	0.1777 (4)	0.1399 (3)	0.4680 (2)	0.0430 (8)	
C3	0.1519 (5)	0.2001 (3)	0.5227 (3)	0.0496 (9)	
C4	0.1912 (7)	0.1635 (4)	0.6053 (3)	0.0677 (13)	
H4A	0.1730	0.2045	0.6399	0.081*	
C5	0.2583 (8)	0.0656 (4)	0.6393 (3)	0.0790 (15)	
H5A	0.2850	0.0422	0.6960	0.095*	
C6	0.2848 (8)	0.0045 (4)	0.5904 (3)	0.0767 (15)	
H6A	0.3314	−0.0605	0.6134	0.092*	
C7	0.2606 (6)	−0.0264 (3)	0.4566 (3)	0.0584 (11)	
H7A	0.3139	−0.0896	0.4820	0.070*	
C8	0.0566 (13)	0.3582 (4)	0.5352 (5)	0.157 (4)	
H8A	0.1019	0.3251	0.5900	0.235*	
H8B	−0.0657	0.3801	0.5402	0.235*	
H8C	0.1133	0.4130	0.5100	0.235*	
C9	0.2341 (5)	−0.0742 (3)	0.3300 (2)	0.0490 (9)	
C10	0.1478 (6)	−0.0465 (3)	0.2556 (3)	0.0660 (12)	
H10A	0.0742	0.0129	0.2396	0.079*	
C11	0.1715 (7)	−0.1076 (4)	0.2050 (3)	0.0713 (13)	
H11A	0.1135	−0.0887	0.1545	0.086*	
C12	0.2790 (6)	−0.1961 (3)	0.2273 (3)	0.0658 (12)	
C13	0.3599 (6)	−0.2229 (3)	0.3029 (3)	0.0736 (14)	
H13A	0.4298	−0.2834	0.3198	0.088*	
C14	0.3409 (6)	−0.1629 (3)	0.3545 (3)	0.0656 (12)	
H14A	0.3991	−0.1820	0.4049	0.079*	
C15	0.3088 (8)	−0.2618 (4)	0.1697 (4)	0.0961 (19)	
H15A	0.3878	−0.3198	0.1952	0.144*	
H15B	0.3569	−0.2286	0.1166	0.144*	
H15C	0.2005	−0.2784	0.1608	0.144*	
C16	0.1935 (5)	0.5007 (3)	0.0389 (2)	0.0442 (8)	
C17	0.1748 (5)	0.4272 (3)	0.1145 (2)	0.0444 (8)	
C18	0.2789 (5)	0.3341 (3)	0.1226 (3)	0.0515 (9)	
C19	0.3874 (5)	0.3167 (3)	0.0591 (3)	0.0584 (11)	
H19A	0.4546	0.2557	0.0658	0.070*	
C20	0.3990 (6)	0.3895 (4)	−0.0160 (3)	0.0622 (12)	
H20A	0.4723	0.3761	−0.0594	0.075*	
C21	0.3052 (6)	0.4793 (4)	−0.0266 (3)	0.0573 (10)	
H21A	0.3141	0.5271	−0.0771	0.069*	
C22	0.1060 (5)	0.5958 (3)	0.0278 (2)	0.0486 (9)	
H22A	0.1260	0.6417	−0.0225	0.058*	
C23	0.3870 (7)	0.1844 (4)	0.2236 (4)	0.102 (2)	
H23A	0.4622	0.1807	0.1758	0.152*	
H23B	0.3339	0.1274	0.2423	0.152*	
H23C	0.4531	0.1885	0.2682	0.152*	
C24	−0.0870 (5)	0.7203 (3)	0.0786 (2)	0.0497 (9)	
C25	−0.1857 (7)	0.7372 (3)	0.1446 (3)	0.0755 (14)	
H25A	−0.1997	0.6857	0.1917	0.091*	
C26	−0.2661 (7)	0.8298 (4)	0.1431 (4)	0.0835 (16)	
H26A	−0.3341	0.8394	0.1893	0.100*	
C27	−0.2489 (7)	0.9068 (4)	0.0766 (4)	0.0710 (14)	
C28	−0.1476 (10)	0.8887 (4)	0.0102 (4)	0.101 (2)	
H28A	−0.1329	0.9405	−0.0365	0.122*	
C29	−0.0668 (8)	0.7971 (4)	0.0099 (3)	0.0880 (17)	
H29A	0.0009	0.7874	−0.0363	0.106*	
C30	−0.3378 (9)	1.0081 (4)	0.0774 (4)	0.102 (2)	
H30A	−0.3124	1.0539	0.0253	0.154*	
H30B	−0.2962	1.0245	0.1233	0.154*	
H30C	−0.4607	1.0098	0.0841	0.154*	
C31	0.3062 (10)	0.4819 (5)	0.2976 (6)	0.135 (3)	
H31A	0.4149	0.4803	0.3211	0.202*	
H31B	0.2204	0.5308	0.3121	0.202*	
H31C	0.3194	0.4967	0.2374	0.202*	
N1	0.2109 (4)	−0.0076 (2)	0.38071 (19)	0.0497 (8)	
H1A	0.1587	0.0504	0.3580	0.060*	
N2	−0.0023 (4)	0.6249 (2)	0.08322 (19)	0.0477 (7)	
H2A	−0.0244	0.5805	0.1277	0.057*	
N3	−0.2286 (6)	0.5186 (3)	0.3175 (3)	0.0749 (11)	
N4	−0.1340 (4)	0.2119 (3)	0.2005 (2)	0.0527 (8)	
N5	−0.3505 (4)	0.2705 (3)	0.3938 (2)	0.0584 (9)	
O1	0.1453 (3)	0.17537 (18)	0.38872 (16)	0.0500 (6)	
O2	0.0872 (4)	0.2928 (2)	0.4830 (2)	0.0655 (8)	
O3	0.0685 (3)	0.44247 (19)	0.17475 (16)	0.0530 (7)	
O4	0.2566 (4)	0.2677 (2)	0.2006 (2)	0.0649 (8)	
O5	0.2525 (5)	0.3889 (3)	0.3306 (3)	0.0876 (12)	
O6	−0.106 (2)	0.4788 (12)	0.3527 (12)	0.106 (5)	0.50
O6'	−0.167 (2)	0.4474 (11)	0.3858 (10)	0.089 (4)	0.50
O7	−0.2290 (6)	0.4991 (3)	0.2523 (3)	0.1052 (14)	
O8	−0.3191 (6)	0.5908 (3)	0.3299 (3)	0.1177 (16)	
O9	−0.0716 (4)	0.1719 (2)	0.27132 (18)	0.0626 (8)	
O10	−0.1404 (4)	0.3005 (2)	0.17246 (17)	0.0638 (8)	
O11	−0.1851 (5)	0.1639 (3)	0.1598 (2)	0.0792 (10)	
O12	−0.2125 (4)	0.2409 (3)	0.4287 (2)	0.0825 (11)	
O13	−0.3464 (4)	0.3214 (3)	0.3193 (2)	0.0868 (11)	
O14	−0.4881 (4)	0.2489 (3)	0.4293 (2)	0.0920 (12)	
H5	0.250 (13)	0.368 (8)	0.384 (6)	0.22 (5)*	

### Atomic displacement parameters (Å^2^)

**Table:**

	*U*^11^	*U*^22^	*U*^33^	*U*^12^	*U*^13^	*U*^23^
Ce	0.03685 (14)	0.03142 (14)	0.05200 (15)	−0.00101 (9)	−0.00009 (9)	−0.00664 (9)
C1	0.061 (2)	0.042 (2)	0.047 (2)	−0.0044 (18)	0.0017 (18)	−0.0072 (17)
C2	0.0358 (19)	0.044 (2)	0.047 (2)	−0.0052 (16)	−0.0008 (15)	−0.0107 (16)
C3	0.045 (2)	0.046 (2)	0.059 (2)	−0.0093 (17)	−0.0015 (17)	−0.0175 (18)
C4	0.081 (3)	0.069 (3)	0.059 (3)	−0.011 (3)	0.000 (2)	−0.029 (2)
C5	0.117 (4)	0.067 (3)	0.047 (2)	−0.009 (3)	−0.011 (3)	−0.009 (2)
C6	0.111 (4)	0.051 (3)	0.057 (3)	−0.001 (3)	−0.015 (3)	−0.002 (2)
C7	0.071 (3)	0.037 (2)	0.057 (2)	0.003 (2)	0.003 (2)	−0.0060 (18)
C8	0.261 (11)	0.061 (4)	0.158 (7)	0.041 (5)	−0.108 (7)	−0.063 (4)
C9	0.051 (2)	0.037 (2)	0.057 (2)	−0.0017 (17)	0.0024 (17)	−0.0148 (17)
C10	0.075 (3)	0.039 (2)	0.077 (3)	0.009 (2)	−0.018 (2)	−0.014 (2)
C11	0.082 (3)	0.061 (3)	0.074 (3)	0.000 (2)	−0.021 (2)	−0.027 (2)
C12	0.058 (3)	0.061 (3)	0.088 (3)	−0.002 (2)	−0.004 (2)	−0.040 (3)
C13	0.076 (3)	0.050 (3)	0.092 (3)	0.017 (2)	−0.013 (3)	−0.029 (2)
C14	0.070 (3)	0.053 (3)	0.068 (3)	0.015 (2)	−0.010 (2)	−0.022 (2)
C15	0.100 (4)	0.084 (4)	0.122 (5)	0.008 (3)	−0.013 (4)	−0.068 (4)
C16	0.0365 (19)	0.050 (2)	0.051 (2)	−0.0142 (16)	0.0010 (15)	−0.0184 (18)
C17	0.0341 (19)	0.050 (2)	0.053 (2)	−0.0097 (16)	0.0038 (15)	−0.0198 (18)
C18	0.043 (2)	0.045 (2)	0.066 (2)	−0.0093 (17)	0.0076 (18)	−0.0157 (19)
C19	0.051 (2)	0.053 (3)	0.077 (3)	−0.0066 (19)	0.010 (2)	−0.031 (2)
C20	0.057 (3)	0.075 (3)	0.066 (3)	−0.018 (2)	0.018 (2)	−0.039 (2)
C21	0.057 (3)	0.067 (3)	0.052 (2)	−0.018 (2)	0.0058 (19)	−0.020 (2)
C22	0.050 (2)	0.051 (2)	0.046 (2)	−0.0148 (18)	−0.0031 (17)	−0.0100 (17)
C23	0.079 (4)	0.067 (4)	0.118 (5)	0.019 (3)	0.026 (3)	0.011 (3)
C24	0.053 (2)	0.044 (2)	0.055 (2)	−0.0113 (18)	−0.0052 (18)	−0.0151 (18)
C25	0.087 (4)	0.049 (3)	0.083 (3)	−0.013 (2)	0.026 (3)	−0.014 (2)
C26	0.086 (4)	0.066 (3)	0.102 (4)	−0.012 (3)	0.025 (3)	−0.037 (3)
C27	0.073 (3)	0.047 (3)	0.097 (4)	−0.004 (2)	−0.020 (3)	−0.027 (3)
C28	0.168 (7)	0.049 (3)	0.077 (4)	−0.004 (4)	−0.005 (4)	−0.011 (3)
C29	0.134 (5)	0.053 (3)	0.064 (3)	−0.002 (3)	0.019 (3)	−0.010 (2)
C30	0.120 (5)	0.059 (3)	0.143 (6)	0.001 (3)	−0.021 (4)	−0.055 (4)
C31	0.115 (6)	0.082 (5)	0.206 (9)	−0.045 (4)	−0.055 (6)	−0.007 (5)
N1	0.055 (2)	0.0361 (17)	0.0513 (18)	0.0040 (14)	0.0015 (15)	−0.0102 (14)
N2	0.0536 (19)	0.0395 (18)	0.0484 (17)	−0.0127 (14)	0.0001 (14)	−0.0073 (14)
N3	0.065 (3)	0.049 (2)	0.105 (3)	0.012 (2)	−0.002 (2)	−0.026 (2)
N4	0.0447 (18)	0.060 (2)	0.0535 (19)	−0.0053 (16)	0.0023 (15)	−0.0188 (17)
N5	0.045 (2)	0.055 (2)	0.059 (2)	−0.0079 (16)	−0.0030 (16)	0.0081 (16)
O1	0.0543 (16)	0.0389 (14)	0.0517 (15)	0.0014 (12)	−0.0093 (12)	−0.0086 (12)
O2	0.079 (2)	0.0424 (16)	0.077 (2)	0.0012 (15)	−0.0212 (16)	−0.0218 (14)
O3	0.0495 (15)	0.0424 (15)	0.0577 (15)	0.0011 (12)	0.0107 (12)	−0.0084 (12)
O4	0.0538 (17)	0.0390 (16)	0.085 (2)	0.0047 (13)	0.0194 (15)	−0.0052 (14)
O5	0.065 (2)	0.076 (3)	0.109 (3)	−0.0266 (19)	−0.028 (2)	0.010 (2)
O6	0.088 (11)	0.089 (12)	0.150 (15)	0.039 (8)	−0.042 (9)	−0.071 (10)
O6'	0.099 (11)	0.064 (8)	0.109 (10)	0.023 (6)	−0.039 (7)	−0.046 (7)
O7	0.122 (3)	0.083 (3)	0.090 (3)	0.021 (2)	0.018 (2)	−0.022 (2)
O8	0.119 (3)	0.078 (3)	0.145 (4)	0.050 (3)	−0.010 (3)	−0.053 (3)
O9	0.075 (2)	0.0462 (17)	0.0642 (18)	−0.0021 (15)	−0.0192 (15)	−0.0129 (14)
O10	0.080 (2)	0.0519 (19)	0.0511 (16)	−0.0078 (16)	−0.0051 (14)	−0.0029 (13)
O11	0.079 (2)	0.097 (3)	0.081 (2)	−0.024 (2)	−0.0089 (18)	−0.047 (2)
O12	0.054 (2)	0.101 (3)	0.078 (2)	−0.0162 (19)	−0.0027 (17)	−0.0003 (19)
O13	0.063 (2)	0.106 (3)	0.073 (2)	−0.0082 (19)	−0.0020 (17)	0.000 (2)
O14	0.0410 (18)	0.111 (3)	0.097 (3)	−0.0190 (18)	0.0048 (17)	0.015 (2)

### Geometric parameters (Å, °)

**Table:**

Ce—O1	2.400 (2)	C17—O3	1.295 (4)
Ce—O3	2.451 (3)	C17—C18	1.429 (5)
Ce—O5	2.509 (4)	C18—C19	1.356 (6)
Ce—O6	2.542 (18)	C18—O4	1.394 (5)
Ce—O6'	2.599 (17)	C19—C20	1.395 (7)
Ce—O9	2.620 (3)	C19—H19A	0.9300
Ce—O12	2.650 (4)	C20—C21	1.352 (6)
Ce—O13	2.650 (3)	C20—H20A	0.9300
Ce—O10	2.661 (3)	C21—H21A	0.9300
Ce—O7	2.733 (4)	C22—N2	1.313 (5)
Ce—O4	2.790 (3)	C22—H22A	0.9300
Ce—O2	2.981 (3)	C23—O4	1.418 (5)
C1—C7	1.394 (6)	C23—H23A	0.9600
C1—C6	1.421 (6)	C23—H23B	0.9600
C1—C2	1.427 (5)	C23—H23C	0.9600
C2—O1	1.294 (4)	C24—C25	1.349 (6)
C2—C3	1.418 (5)	C24—C29	1.372 (6)
C3—O2	1.345 (5)	C24—N2	1.413 (5)
C3—C4	1.359 (6)	C25—C26	1.379 (7)
C4—C5	1.394 (7)	C25—H25A	0.9300
C4—H4A	0.9300	C26—C27	1.348 (8)
C5—C6	1.352 (7)	C26—H26A	0.9300
C5—H5A	0.9300	C27—C28	1.369 (8)
C6—H6A	0.9300	C27—C30	1.520 (7)
C7—N1	1.286 (5)	C28—C29	1.373 (7)
C7—H7A	0.9300	C28—H28A	0.9300
C8—O2	1.446 (6)	C29—H29A	0.9300
C8—H8A	0.9600	C30—H30A	0.9600
C8—H8B	0.9600	C30—H30B	0.9600
C8—H8C	0.9600	C30—H30C	0.9600
C9—C10	1.376 (6)	C31—O5	1.439 (7)
C9—C14	1.378 (5)	C31—H31A	0.9600
C9—N1	1.442 (5)	C31—H31B	0.9600
C10—C11	1.375 (6)	C31—H31C	0.9600
C10—H10A	0.9300	N1—H1A	0.8600
C11—C12	1.375 (6)	N2—H2A	0.8600
C11—H11A	0.9300	N3—O6	1.135 (19)
C12—C13	1.373 (7)	N3—O7	1.198 (6)
C12—C15	1.520 (6)	N3—O8	1.221 (5)
C13—C14	1.377 (6)	N3—O6'	1.348 (17)
C13—H13A	0.9300	N4—O10	1.236 (4)
C14—H14A	0.9300	N4—O11	1.238 (4)
C15—H15A	0.9600	N4—O9	1.246 (4)
C15—H15B	0.9600	N5—O12	1.215 (4)
C15—H15C	0.9600	N5—O14	1.238 (4)
C16—C22	1.399 (5)	N5—O13	1.247 (5)
C16—C17	1.413 (5)	O5—H5	0.85 (10)
C16—C21	1.414 (6)		
			
O1—Ce—O3	131.99 (9)	C12—C13—C14	122.2 (4)
O1—Ce—O5	82.46 (11)	C12—C13—H13A	118.9
O3—Ce—O5	71.49 (12)	C14—C13—H13A	118.9
O1—Ce—O6	125.8 (4)	C13—C14—C9	118.7 (4)
O3—Ce—O6	82.9 (4)	C13—C14—H14A	120.7
O5—Ce—O6	70.8 (4)	C9—C14—H14A	120.7
O1—Ce—O6'	116.9 (4)	C12—C15—H15A	109.5
O3—Ce—O6'	99.3 (3)	C12—C15—H15B	109.5
O5—Ce—O6'	82.7 (4)	H15A—C15—H15B	109.5
O6—Ce—O6'	17.8 (4)	C12—C15—H15C	109.5
O1—Ce—O9	65.07 (9)	H15A—C15—H15C	109.5
O3—Ce—O9	109.10 (9)	H15B—C15—H15C	109.5
O5—Ce—O9	136.63 (13)	C22—C16—C17	121.2 (3)
O6—Ce—O9	151.9 (4)	C22—C16—C21	118.7 (4)
O6'—Ce—O9	136.8 (4)	C17—C16—C21	120.0 (4)
O1—Ce—O12	69.58 (10)	O3—C17—C16	122.1 (3)
O3—Ce—O12	157.78 (10)	O3—C17—C18	120.5 (4)
O5—Ce—O12	123.22 (14)	C16—C17—C18	117.3 (3)
O6—Ce—O12	86.8 (4)	C19—C18—O4	125.9 (4)
O6'—Ce—O12	69.1 (3)	C19—C18—C17	121.0 (4)
O9—Ce—O12	72.70 (12)	O4—C18—C17	113.1 (3)
O1—Ce—O13	109.50 (11)	C18—C19—C20	120.6 (4)
O3—Ce—O13	112.21 (10)	C18—C19—H19A	119.7
O5—Ce—O13	152.32 (16)	C20—C19—H19A	119.7
O6—Ce—O13	82.3 (4)	C21—C20—C19	120.9 (4)
O6'—Ce—O13	69.6 (4)	C21—C20—H20A	119.6
O9—Ce—O13	69.74 (12)	C19—C20—H20A	119.6
O12—Ce—O13	46.60 (10)	C20—C21—C16	120.1 (4)
O1—Ce—O10	110.07 (9)	C20—C21—H21A	119.9
O3—Ce—O10	68.54 (10)	C16—C21—H21A	119.9
O5—Ce—O10	135.05 (13)	N2—C22—C16	124.7 (4)
O6—Ce—O10	122.3 (4)	N2—C22—H22A	117.6
O6'—Ce—O10	123.1 (4)	C16—C22—H22A	117.6
O9—Ce—O10	47.34 (9)	O4—C23—H23A	109.5
O12—Ce—O10	101.26 (11)	O4—C23—H23B	109.5
O13—Ce—O10	65.67 (11)	H23A—C23—H23B	109.5
O1—Ce—O7	164.33 (12)	O4—C23—H23C	109.5
O3—Ce—O7	62.27 (11)	H23A—C23—H23C	109.5
O5—Ce—O7	98.96 (15)	H23B—C23—H23C	109.5
O6—Ce—O7	42.4 (5)	C25—C24—C29	118.8 (4)
O6'—Ce—O7	48.4 (4)	C25—C24—N2	119.3 (4)
O9—Ce—O7	120.16 (13)	C29—C24—N2	121.9 (4)
O12—Ce—O7	97.17 (13)	C24—C25—C26	120.8 (5)
O13—Ce—O7	62.84 (14)	C24—C25—H25A	119.6
O10—Ce—O7	80.03 (13)	C26—C25—H25A	119.6
O1—Ce—O4	74.23 (9)	C27—C26—C25	121.9 (5)
O3—Ce—O4	60.05 (8)	C27—C26—H26A	119.1
O5—Ce—O4	73.09 (14)	C25—C26—H26A	119.1
O6—Ce—O4	134.6 (4)	C26—C27—C28	116.7 (5)
O6'—Ce—O4	152.0 (3)	C26—C27—C30	120.5 (5)
O9—Ce—O4	70.99 (10)	C28—C27—C30	122.8 (5)
O12—Ce—O4	136.71 (11)	C27—C28—C29	122.6 (5)
O13—Ce—O4	133.55 (11)	C27—C28—H28A	118.7
O10—Ce—O4	69.73 (10)	C29—C28—H28A	118.7
O7—Ce—O4	121.21 (11)	C24—C29—C28	119.3 (5)
O1—Ce—O2	56.65 (8)	C24—C29—H29A	120.4
O3—Ce—O2	131.21 (9)	C28—C29—H29A	120.4
O5—Ce—O2	61.99 (13)	C27—C30—H30A	109.5
O6—Ce—O2	69.2 (4)	C27—C30—H30B	109.5
O6'—Ce—O2	62.3 (4)	H30A—C30—H30B	109.5
O9—Ce—O2	114.34 (8)	C27—C30—H30C	109.5
O12—Ce—O2	61.31 (10)	H30A—C30—H30C	109.5
O13—Ce—O2	102.94 (10)	H30B—C30—H30C	109.5
O10—Ce—O2	160.09 (10)	O5—C31—H31A	109.5
O7—Ce—O2	110.16 (11)	O5—C31—H31B	109.5
O4—Ce—O2	115.27 (9)	H31A—C31—H31B	109.5
C7—C1—C6	118.8 (4)	O5—C31—H31C	109.5
C7—C1—C2	121.3 (4)	H31A—C31—H31C	109.5
C6—C1—C2	119.9 (4)	H31B—C31—H31C	109.5
O1—C2—C3	121.1 (3)	C7—N1—C9	126.4 (3)
O1—C2—C1	121.8 (3)	C7—N1—H1A	116.8
C3—C2—C1	117.2 (3)	C9—N1—H1A	116.8
O2—C3—C4	126.5 (4)	C22—N2—C24	127.9 (3)
O2—C3—C2	112.5 (3)	C22—N2—H2A	116.1
C4—C3—C2	121.1 (4)	C24—N2—H2A	116.1
C3—C4—C5	121.1 (4)	O6—N3—O7	110.2 (10)
C3—C4—H4A	119.4	O6—N3—O8	124.5 (10)
C5—C4—H4A	119.4	O7—N3—O8	122.0 (5)
C6—C5—C4	120.5 (4)	O6—N3—O6'	36.2 (10)
C6—C5—H5A	119.7	O7—N3—O6'	118.3 (8)
C4—C5—H5A	119.7	O8—N3—O6'	117.0 (8)
C5—C6—C1	120.1 (4)	O10—N4—O11	121.8 (4)
C5—C6—H6A	119.9	O10—N4—O9	117.4 (3)
C1—C6—H6A	119.9	O11—N4—O9	120.8 (4)
N1—C7—C1	125.8 (4)	O12—N5—O14	122.0 (4)
N1—C7—H7A	117.1	O12—N5—O13	116.7 (4)
C1—C7—H7A	117.1	O14—N5—O13	121.3 (3)
O2—C8—H8A	109.5	C2—O1—Ce	133.8 (2)
O2—C8—H8B	109.5	C3—O2—C8	115.4 (4)
H8A—C8—H8B	109.5	C3—O2—Ce	114.4 (2)
O2—C8—H8C	109.5	C8—O2—Ce	129.7 (3)
H8A—C8—H8C	109.5	C17—O3—Ce	129.0 (2)
H8B—C8—H8C	109.5	C18—O4—C23	116.6 (3)
C10—C9—C14	120.4 (4)	C18—O4—Ce	116.5 (2)
C10—C9—N1	118.3 (3)	C23—O4—Ce	126.2 (3)
C14—C9—N1	121.3 (4)	C31—O5—Ce	132.0 (4)
C11—C10—C9	119.4 (4)	C31—O5—H5	115 (7)
C11—C10—H10A	120.3	Ce—O5—H5	97 (7)
C9—C10—H10A	120.3	N3—O6—Ce	106.5 (12)
C10—C11—C12	121.5 (4)	N3—O6'—Ce	96.7 (8)
C10—C11—H11A	119.2	N3—O7—Ce	94.3 (3)
C12—C11—H11A	119.2	N4—O9—Ce	98.5 (2)
C13—C12—C11	117.8 (4)	N4—O10—Ce	96.8 (2)
C13—C12—C15	121.0 (4)	N5—O12—Ce	98.8 (3)
C11—C12—C15	121.1 (5)	N5—O13—Ce	97.8 (2)
			
C7—C1—C2—O1	−4.6 (6)	O13—Ce—O4—C18	85.3 (3)
C6—C1—C2—O1	176.5 (4)	O10—Ce—O4—C18	68.5 (3)
C7—C1—C2—C3	175.8 (4)	O7—Ce—O4—C18	4.6 (3)
C6—C1—C2—C3	−3.2 (6)	O2—Ce—O4—C18	−132.4 (3)
O1—C2—C3—O2	2.0 (5)	O1—Ce—O4—C23	−3.0 (4)
C1—C2—C3—O2	−178.4 (3)	O3—Ce—O4—C23	161.8 (5)
O1—C2—C3—C4	−177.8 (4)	O5—Ce—O4—C23	83.7 (4)
C1—C2—C3—C4	1.8 (6)	O6—Ce—O4—C23	122.1 (7)
O2—C3—C4—C5	−179.8 (5)	O6'—Ce—O4—C23	115.1 (9)
C2—C3—C4—C5	0.0 (7)	O9—Ce—O4—C23	−71.5 (4)
C3—C4—C5—C6	−0.4 (9)	O12—Ce—O4—C23	−37.1 (5)
C4—C5—C6—C1	−1.0 (9)	O13—Ce—O4—C23	−105.1 (4)
C7—C1—C6—C5	−176.1 (5)	O10—Ce—O4—C23	−122.0 (4)
C2—C1—C6—C5	2.9 (8)	O7—Ce—O4—C23	174.1 (4)
C6—C1—C7—N1	172.6 (5)	O2—Ce—O4—C23	37.1 (4)
C2—C1—C7—N1	−6.4 (7)	O1—Ce—O5—C31	172.8 (7)
C14—C9—C10—C11	−1.1 (8)	O3—Ce—O5—C31	33.7 (7)
N1—C9—C10—C11	178.2 (4)	O6—Ce—O5—C31	−55.0 (8)
C9—C10—C11—C12	0.3 (8)	O6'—Ce—O5—C31	−68.6 (8)
C10—C11—C12—C13	1.3 (8)	O9—Ce—O5—C31	132.2 (6)
C10—C11—C12—C15	−178.0 (6)	O12—Ce—O5—C31	−127.7 (7)
C11—C12—C13—C14	−2.2 (9)	O13—Ce—O5—C31	−69.0 (8)
C15—C12—C13—C14	177.1 (6)	O10—Ce—O5—C31	61.9 (7)
C12—C13—C14—C9	1.4 (8)	O7—Ce—O5—C31	−22.9 (7)
C10—C9—C14—C13	0.3 (8)	O4—Ce—O5—C31	97.1 (7)
N1—C9—C14—C13	−179.1 (5)	O2—Ce—O5—C31	−131.1 (7)
C22—C16—C17—O3	4.4 (6)	O7—N3—O6—Ce	−26.8 (10)
C21—C16—C17—O3	−177.0 (4)	O8—N3—O6—Ce	173.5 (5)
C22—C16—C17—C18	−175.5 (4)	O6'—N3—O6—Ce	84 (2)
C21—C16—C17—C18	3.2 (5)	O1—Ce—O6—N3	−150.2 (8)
O3—C17—C18—C19	178.4 (4)	O3—Ce—O6—N3	72.0 (10)
C16—C17—C18—C19	−1.8 (6)	O5—Ce—O6—N3	144.8 (11)
O3—C17—C18—O4	−2.4 (5)	O6'—Ce—O6—N3	−85 (3)
C16—C17—C18—O4	177.5 (3)	O9—Ce—O6—N3	−45.8 (16)
O4—C18—C19—C20	−179.6 (4)	O12—Ce—O6—N3	−88.3 (10)
C17—C18—C19—C20	−0.4 (6)	O13—Ce—O6—N3	−41.6 (9)
C18—C19—C20—C21	1.3 (7)	O10—Ce—O6—N3	13.0 (12)
C19—C20—C21—C16	0.1 (7)	O7—Ce—O6—N3	16.0 (7)
C22—C16—C21—C20	176.3 (4)	O4—Ce—O6—N3	105.9 (10)
C17—C16—C21—C20	−2.4 (6)	O2—Ce—O6—N3	−148.7 (11)
C17—C16—C22—N2	−2.1 (6)	O6—N3—O6'—Ce	−70 (2)
C21—C16—C22—N2	179.3 (4)	O7—N3—O6'—Ce	15.9 (9)
C29—C24—C25—C26	0.5 (8)	O8—N3—O6'—Ce	177.8 (4)
N2—C24—C25—C26	178.2 (5)	O1—Ce—O6'—N3	178.4 (6)
C24—C25—C26—C27	−0.4 (9)	O3—Ce—O6'—N3	31.0 (8)
C25—C26—C27—C28	0.1 (9)	O5—Ce—O6'—N3	100.8 (8)
C25—C26—C27—C30	−179.3 (5)	O6—Ce—O6'—N3	54 (2)
C26—C27—C28—C29	0.3 (10)	O9—Ce—O6'—N3	−100.2 (7)
C30—C27—C28—C29	179.6 (6)	O12—Ce—O6'—N3	−129.4 (9)
C25—C24—C29—C28	−0.2 (9)	O13—Ce—O6'—N3	−79.4 (8)
N2—C24—C29—C28	−177.8 (5)	O10—Ce—O6'—N3	−39.4 (9)
C27—C28—C29—C24	−0.2 (10)	O7—Ce—O6'—N3	−8.1 (5)
C1—C7—N1—C9	178.1 (4)	O4—Ce—O6'—N3	70.7 (13)
C10—C9—N1—C7	169.4 (4)	O2—Ce—O6'—N3	162.9 (9)
C14—C9—N1—C7	−11.3 (7)	O6—N3—O7—Ce	23.8 (10)
C16—C22—N2—C24	175.9 (4)	O8—N3—O7—Ce	−175.9 (5)
C25—C24—N2—C22	−175.7 (4)	O6'—N3—O7—Ce	−15.0 (9)
C29—C24—N2—C22	1.9 (7)	O1—Ce—O7—N3	31.4 (7)
C3—C2—O1—Ce	−14.3 (5)	O3—Ce—O7—N3	−126.2 (4)
C1—C2—O1—Ce	166.1 (3)	O5—Ce—O7—N3	−62.7 (4)
O3—Ce—O1—C2	130.1 (3)	O6—Ce—O7—N3	−14.5 (6)
O5—Ce—O1—C2	73.5 (4)	O6'—Ce—O7—N3	9.1 (5)
O6—Ce—O1—C2	13.8 (6)	O9—Ce—O7—N3	136.8 (3)
O6'—Ce—O1—C2	−4.3 (5)	O12—Ce—O7—N3	62.7 (4)
O9—Ce—O1—C2	−136.0 (4)	O13—Ce—O7—N3	95.3 (4)
O12—Ce—O1—C2	−56.2 (3)	O10—Ce—O7—N3	162.9 (4)
O13—Ce—O1—C2	−80.7 (3)	O4—Ce—O7—N3	−138.3 (3)
O10—Ce—O1—C2	−151.1 (3)	O2—Ce—O7—N3	0.7 (4)
O7—Ce—O1—C2	−22.8 (6)	O10—N4—O9—Ce	1.8 (4)
O4—Ce—O1—C2	148.0 (3)	O11—N4—O9—Ce	−179.2 (3)
O2—Ce—O1—C2	12.2 (3)	O1—Ce—O9—N4	−161.6 (3)
C4—C3—O2—C8	−0.5 (8)	O3—Ce—O9—N4	−33.3 (2)
C2—C3—O2—C8	179.7 (6)	O5—Ce—O9—N4	−116.2 (3)
C4—C3—O2—Ce	−173.6 (4)	O6—Ce—O9—N4	78.5 (9)
C2—C3—O2—Ce	6.5 (4)	O6'—Ce—O9—N4	94.9 (6)
O1—Ce—O2—C3	−8.8 (3)	O12—Ce—O9—N4	123.4 (2)
O3—Ce—O2—C3	−128.0 (3)	O13—Ce—O9—N4	74.1 (2)
O5—Ce—O2—C3	−108.7 (3)	O10—Ce—O9—N4	−1.0 (2)
O6—Ce—O2—C3	172.6 (5)	O7—Ce—O9—N4	35.1 (3)
O6'—Ce—O2—C3	154.6 (5)	O4—Ce—O9—N4	−80.6 (2)
O9—Ce—O2—C3	22.8 (3)	O2—Ce—O9—N4	169.6 (2)
O12—Ce—O2—C3	74.6 (3)	O11—N4—O10—Ce	179.3 (3)
O13—Ce—O2—C3	96.2 (3)	O9—N4—O10—Ce	−1.8 (4)
O10—Ce—O2—C3	43.6 (4)	O1—Ce—O10—N4	19.7 (2)
O7—Ce—O2—C3	161.7 (3)	O3—Ce—O10—N4	148.2 (2)
O4—Ce—O2—C3	−56.7 (3)	O5—Ce—O10—N4	119.4 (2)
O1—Ce—O2—C8	179.3 (6)	O6—Ce—O10—N4	−145.8 (5)
O3—Ce—O2—C8	60.1 (6)	O6'—Ce—O10—N4	−124.7 (4)
O5—Ce—O2—C8	79.4 (6)	O9—Ce—O10—N4	1.0 (2)
O6—Ce—O2—C8	0.7 (7)	O12—Ce—O10—N4	−52.4 (2)
O6'—Ce—O2—C8	−17.3 (7)	O13—Ce—O10—N4	−83.2 (2)
O9—Ce—O2—C8	−149.1 (6)	O7—Ce—O10—N4	−147.8 (2)
O12—Ce—O2—C8	−97.4 (6)	O4—Ce—O10—N4	83.4 (2)
O13—Ce—O2—C8	−75.7 (6)	O2—Ce—O10—N4	−25.0 (4)
O10—Ce—O2—C8	−128.4 (6)	O14—N5—O12—Ce	−179.4 (4)
O7—Ce—O2—C8	−10.2 (6)	O13—N5—O12—Ce	2.9 (5)
O4—Ce—O2—C8	131.4 (6)	O1—Ce—O12—N5	−149.0 (3)
C16—C17—O3—Ce	173.8 (2)	O3—Ce—O12—N5	18.4 (5)
C18—C17—O3—Ce	−6.4 (5)	O5—Ce—O12—N5	145.2 (3)
O1—Ce—O3—C17	27.4 (4)	O6—Ce—O12—N5	80.7 (5)
O5—Ce—O3—C17	88.2 (3)	O6'—Ce—O12—N5	79.7 (5)
O6—Ce—O3—C17	160.3 (5)	O9—Ce—O12—N5	−79.8 (3)
O6'—Ce—O3—C17	167.2 (5)	O13—Ce—O12—N5	−1.7 (3)
O9—Ce—O3—C17	−45.8 (3)	O10—Ce—O12—N5	−41.6 (3)
O12—Ce—O3—C17	−136.7 (4)	O7—Ce—O12—N5	39.6 (3)
O13—Ce—O3—C17	−121.1 (3)	O4—Ce—O12—N5	−113.9 (3)
O10—Ce—O3—C17	−70.8 (3)	O2—Ce—O12—N5	148.7 (3)
O7—Ce—O3—C17	−160.6 (4)	O12—N5—O13—Ce	−2.9 (5)
O4—Ce—O3—C17	7.5 (3)	O14—N5—O13—Ce	179.4 (4)
O2—Ce—O3—C17	106.1 (3)	O1—Ce—O13—N5	34.0 (3)
C19—C18—O4—C23	16.8 (7)	O3—Ce—O13—N5	−170.3 (3)
C17—C18—O4—C23	−162.4 (4)	O5—Ce—O13—N5	−78.0 (4)
C19—C18—O4—Ce	−172.6 (3)	O6—Ce—O13—N5	−91.3 (5)
C17—C18—O4—Ce	8.1 (4)	O6'—Ce—O13—N5	−78.4 (5)
O1—Ce—O4—C18	−172.5 (3)	O9—Ce—O13—N5	86.6 (3)
O3—Ce—O4—C18	−7.8 (3)	O12—Ce—O13—N5	1.6 (3)
O5—Ce—O4—C18	−85.8 (3)	O10—Ce—O13—N5	137.9 (3)
O6—Ce—O4—C18	−47.4 (7)	O7—Ce—O13—N5	−131.1 (3)
O6'—Ce—O4—C18	−54.4 (9)	O4—Ce—O13—N5	120.5 (3)
O9—Ce—O4—C18	118.9 (3)	O2—Ce—O13—N5	−24.9 (3)
O12—Ce—O4—C18	153.4 (3)		

### Hydrogen-bond geometry (Å, °)

**Table:**

*D*—H···*A*	*D*—H	H···*A*	*D*···*A*	*D*—H···*A*
C7—H7A···O12^i^	0.93	2.51	3.233 (6)	135
C7—H7A···O14^i^	0.93	2.57	3.488 (5)	169
C30—H30B···O11^ii^	0.96	2.60	3.405 (6)	142
C13—H13A···O8^iii^	0.93	2.42	3.304 (5)	160
C22—H22A···O10^iv^	0.93	2.39	3.243 (5)	153
C29—H29A···O11^iv^	0.93	2.42	3.285 (7)	154
C8—H8B···O6	0.96	3.04	3.26 (2)	94
C10—H10A···O9	0.93	2.57	3.410 (5)	150
C25—H25A···O8	0.93	2.52	3.411 (7)	160
N1—H1A···O1	0.86	2.02	2.671 (4)	132
N1—H1A···O9	0.86	2.50	3.290 (4)	154
N2—H2A···O3	0.86	1.96	2.638 (4)	135
N2—H2A···O7	0.86	2.65	3.444 (6)	154

Symmetry codes: (i) −*x*, −*y*, −*z*+1; (ii) *x*, *y*+1, *z*; (iii) *x*+1, *y*−1, *z*; (iv) −*x*, −*y*+1, −*z*.
